# Supplementary figures and images for: The Potential of ANK1 to Predict Parkinson’s Disease
Source: Genes (Basel). 2023 Jan 15;14(1):226. doi: 10.3390/genes14010226 (PMC9859451; doi:10.3390/genes14010226)

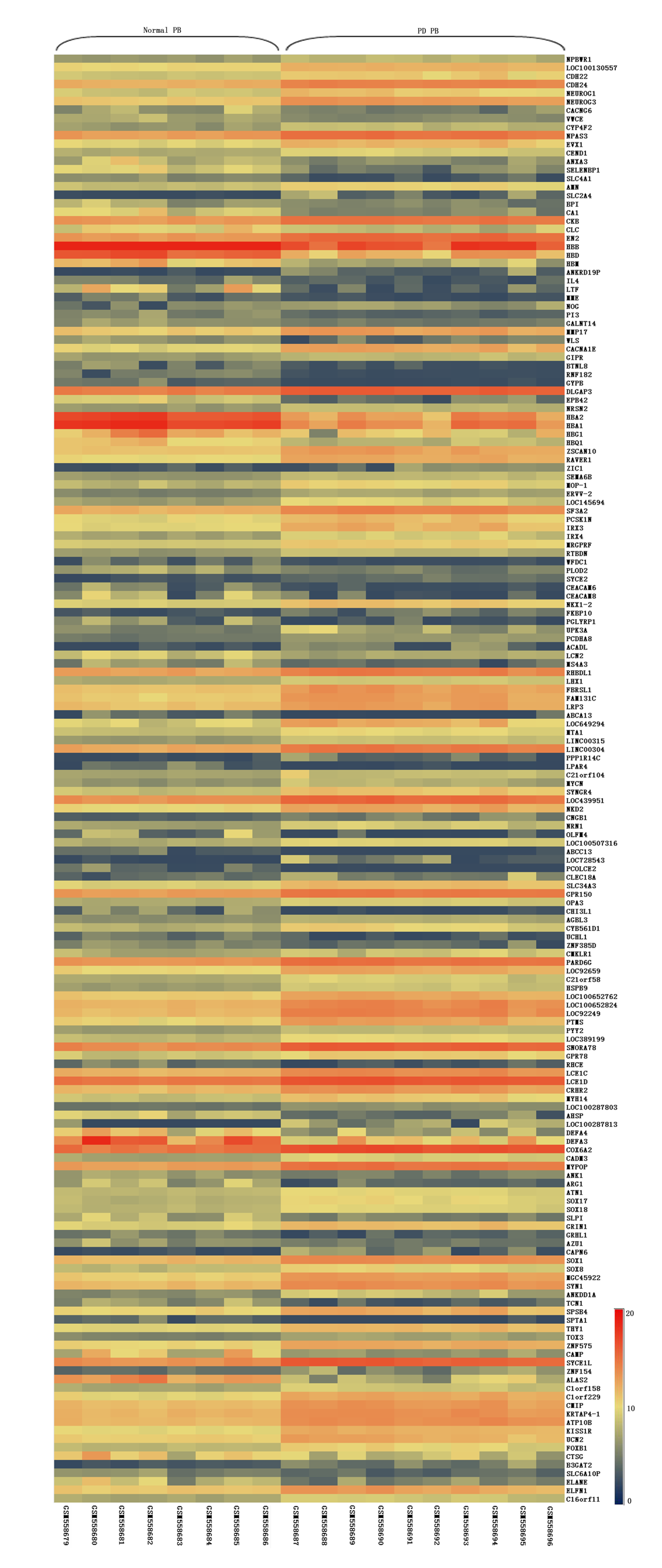

Supplement: Supplementary file 1 [file genes-14-00226-s001.zip › Figure S1.tif]

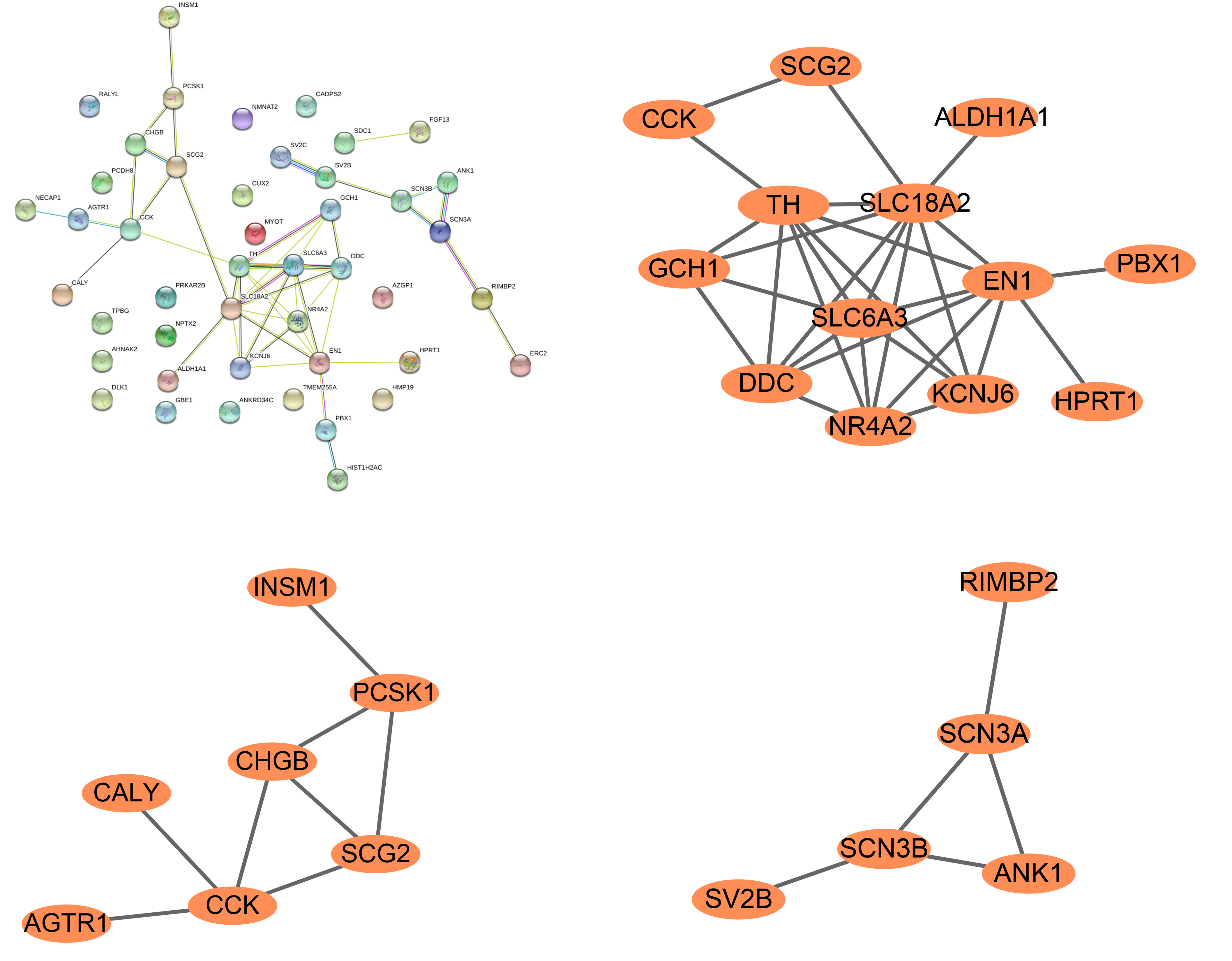

Supplement: Supplementary file 1 [file genes-14-00226-s001.zip › Figure S2.tif]

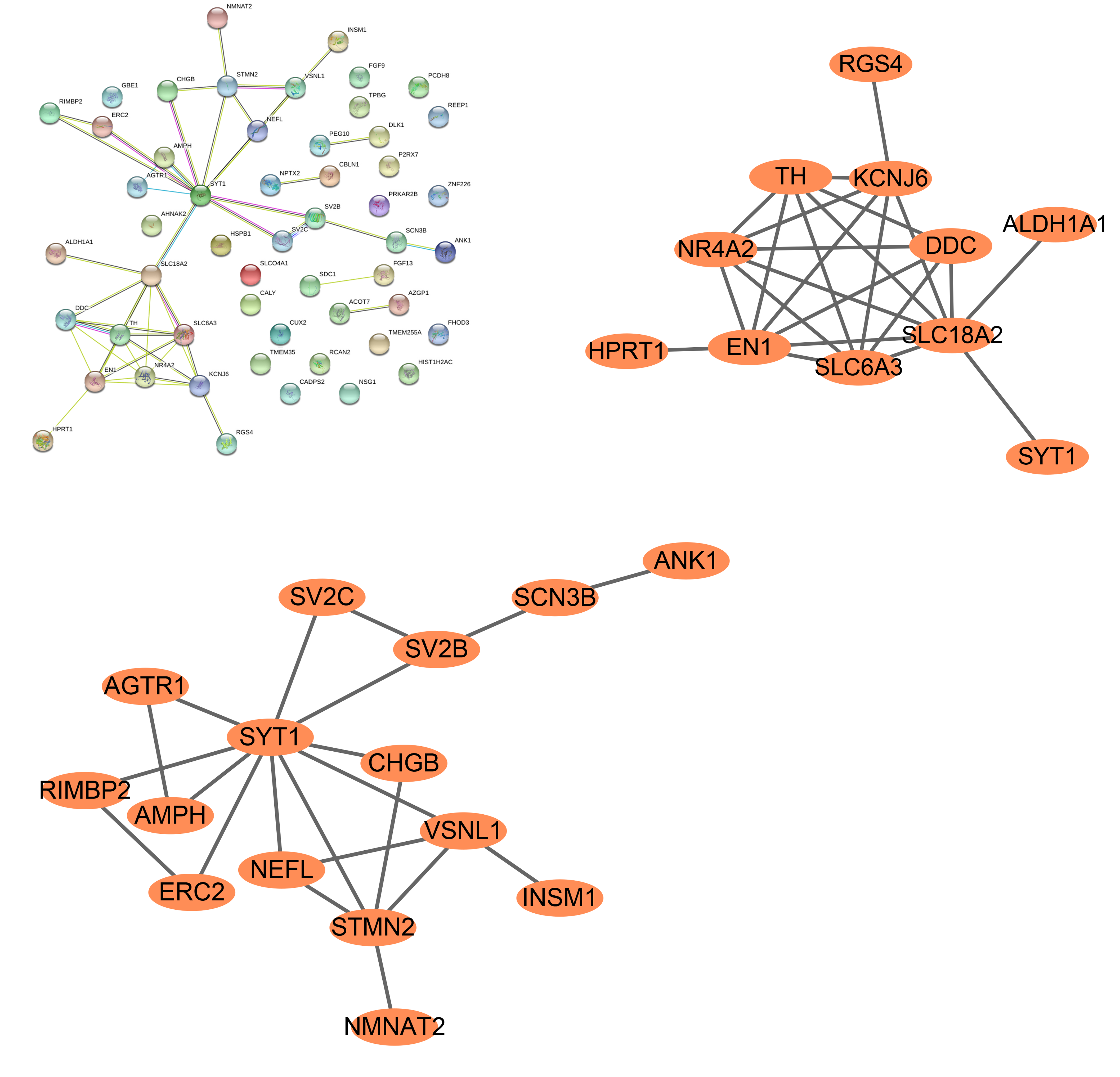

Supplement: Supplementary file 1 [file genes-14-00226-s001.zip › Figure S3.tif]

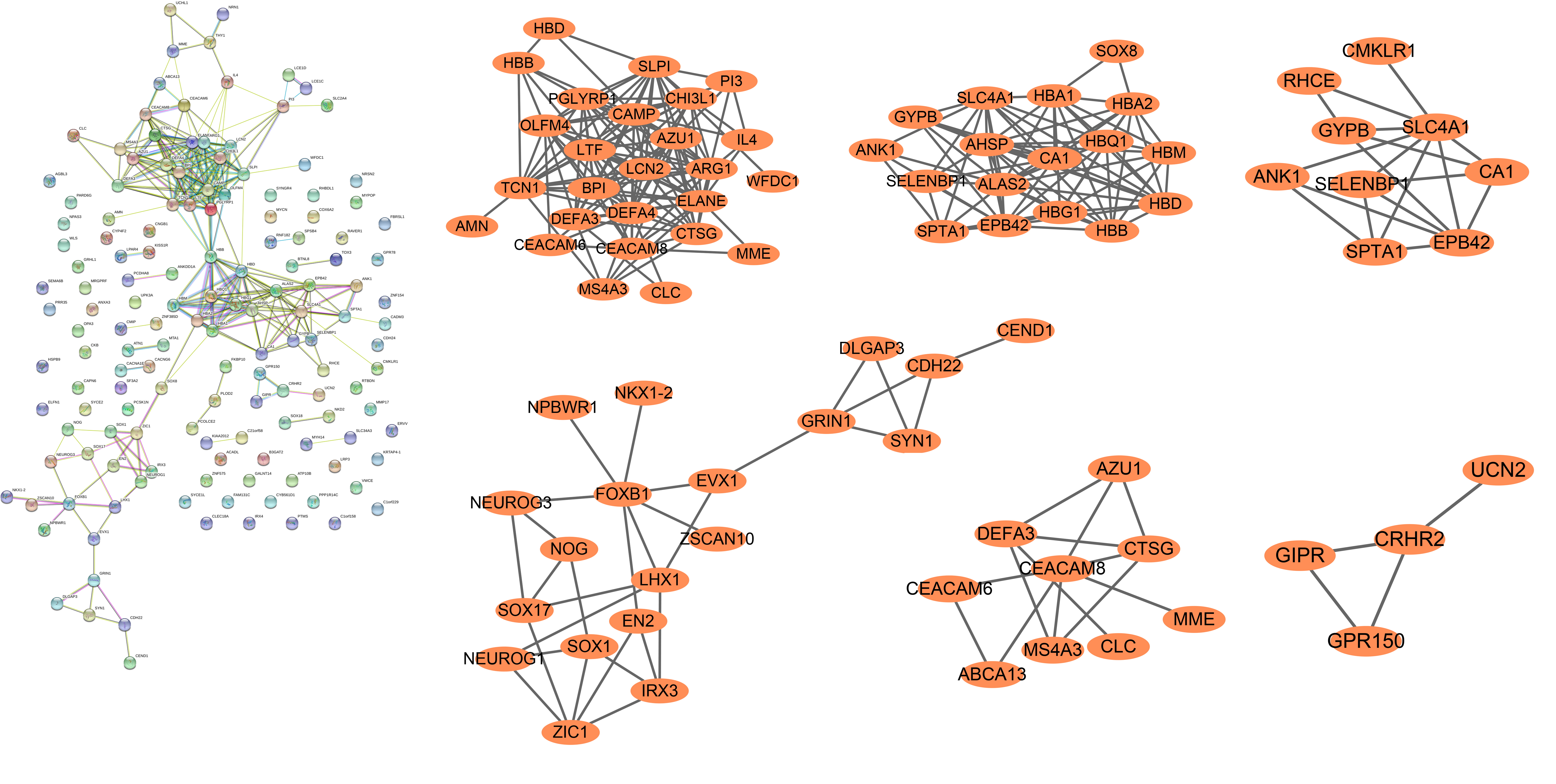

Supplement: Supplementary file 1 [file genes-14-00226-s001.zip › Figure S4.tif]

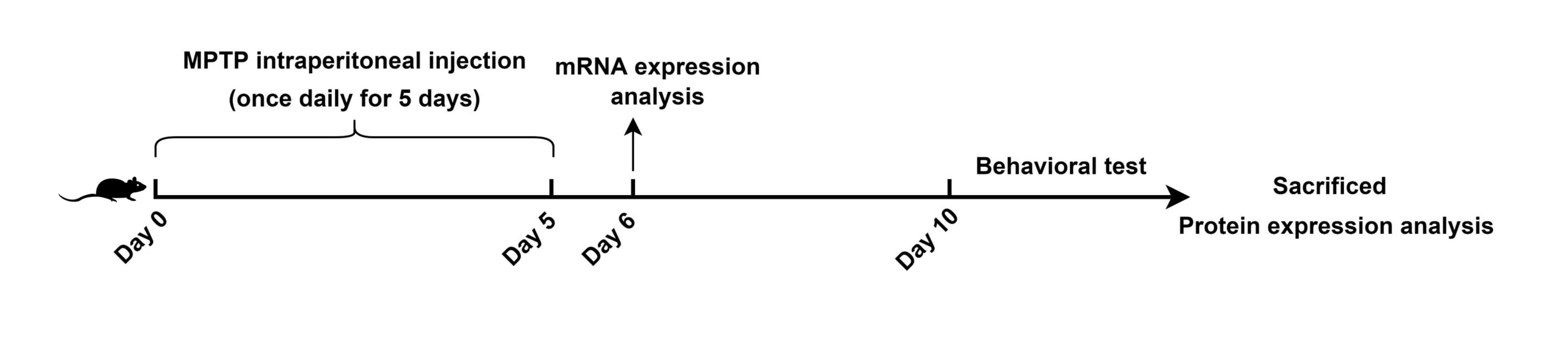

Supplement: Supplementary file 1 [file genes-14-00226-s001.zip › Figure S5.tif]

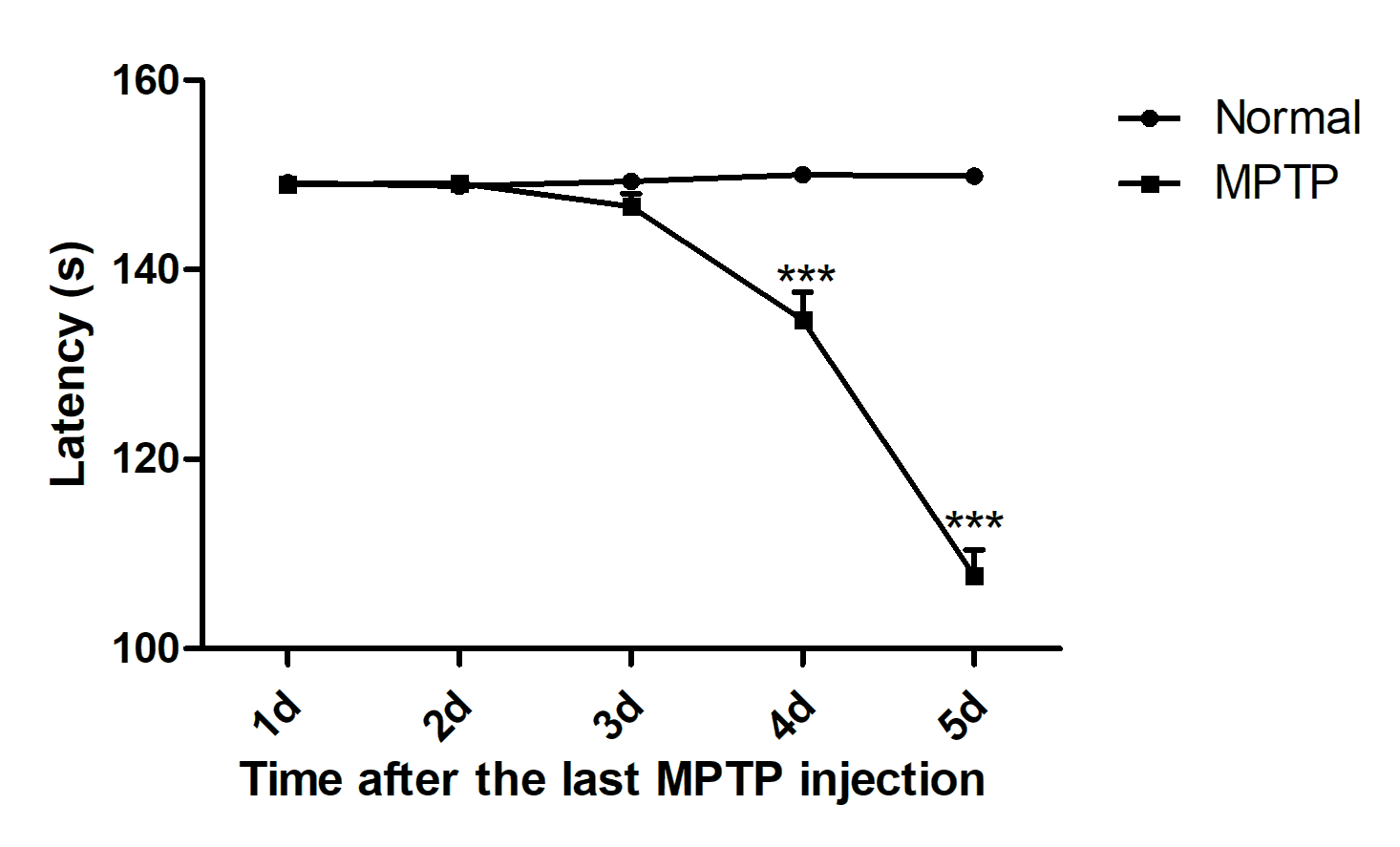

Supplement: Supplementary file 1 [file genes-14-00226-s001.zip › Figure S6.tif]
